# Identification of high lead exposure locations in Ohio at the census tract scale using a generalizable geospatial hotspot approach

**DOI:** 10.1038/s41370-024-00666-x

**Published:** 2024-04-04

**Authors:** Lindsay W. Stanek, Jianping Xue, Valerie G. Zartarian, Antonios G. Poulakos, Rogelio Tornero-Velez, Emily G. Snyder, Alan Walts, Kathy Triantafillou

**Affiliations:** 1 U.S. Environmental Protection Agency (EPA), Office of Research and Development (ORD), Research Triangle Park, North Carolina, NC USA; 2 LinTech Global, Inc., Boston, MA USA; 3 U.S. EPA, Region 5, Chicago, IL USA

**Keywords:** Child Exposure/Health, Metals, Environmental Justice, Geospatial, Analyses

## Abstract

**Background:**

Lead is a persistent, ubiquitous pollutant whose historical sources have been largely addressed through regulation and voluntary actions. The United States (U.S.) has achieved significant decreases in children’s blood lead levels (BLL) over the past 40 years; however, there is no known safe level of Pb exposure. Some communities continue to be disproportionately impacted by exposure to Pb, including Black children and families living in older homes.

**Objective:**

To identify Ohio (OH) census tracts with children exposed to Pb and evaluate potential exposure determinants.

**Methods:**

We obtained individual children’s blood Pb data from 2005–2018 in OH. The percent of children with elevated BLL (EBLL) was calculated for OH census tracts using three blood Pb reference values (3.5, 5, and 10 µg/dL). Getis-Ord Gi* geospatial hotspot or top 20th percentile methodologies were then applied to identify “hotspots.” Findings across multiple time periods and blood Pb reference values were evaluated and compared with existing Pb exposure indices and models.

**Results:**

Consistency was observed across different blood Pb reference values, with the main hotspots identified at 3.5 µg/dL, also identified at 5 and 10 µg/dL. Substantial gains in public health were demonstrated, with the biggest decreases in the number of census tracts with EBLL observed between 2008–2010 and 2011–2013. Across OH, 355 census tracts (of 2850) were identified as hotspots across 17 locations, with the majority in the most populated cites. Generally, old housing and sociodemographic factors were indicators of these EBLL hotspots. A smaller number of hotspots were not associated with these exposure determinants. Variables of race, income, and education level were all strong predictors of hotspots.

**Impact statement:**

The Getis-Ord Gi* geospatial hotspot analysis can inform local investigations into potential Pb exposures for children living in OH. The successful application of a generalizable childhood blood Pb methodology at the census tract scale provides results that are more readily actionable. The moderate agreement of the measured blood Pb results with public Pb indices provide confidence that these indices can be used in the absence of available blood Pb surveillance data. While not a replacement for universal blood Pb testing, a consistent approach can be applied to identify areas where Pb exposure may be problematic.

## Introduction

The United States (U.S.) has made tremendous strides in eliminating many lead (Pb) sources through regulation of industry and consumer products; however, the potential remains for people to come into contact with Pb in their daily lives. Historical reservoirs of environmental Pb exist from decades of emissions and use [1, 2]. Pb is particularly harmful for young children, where Pb exposure can impact neurocognition and behavior at relatively low blood lead levels (BLL) [3–6]. Based on elevated BLL (EBLL) prevalence (≥5 µg/dL) from the National Health and Nutrition Examination Survey (NHANES), over 250,000 children aged 1–5 years in the U.S. are exposed to Pb at levels of potential concern [7]. With the recent lowering of the blood Pb reference value to 3.5 µg/dL by the U.S. Centers for Disease Control and Prevention (CDC) [8], this number is likely to be higher. Older housing may contain contaminated dust from Pb-based paint or drinking water from lead service lines (LSL), making it an important risk factor for EBLL. Furthermore, significant disparities continue to persist in the U.S., with non-Hispanic African American children having higher BLL compared to non-Hispanic white children [9] and those from lower income households having greater prevalence of EBLL compared to higher income levels [10]. There is often overlap between housing, race, income, and other stressors in inner cities, where residents can be at greater risk of being impacted by Pb.

The U.S. government recognizes that Pb remains an important public health issue where more can be done to reduce and eliminate exposures [11, 12]. The U.S. Environmental Protection Agency (EPA) has a critical role to play in this whole of government approach and has committed to take specific actions to prevent childhood Pb exposures, particularly in overburdened communities [13]. One goal of EPA’s strategy is to identify communities with high Pb exposures through science-based approaches using available data, statistical models, and geospatial analyses at census tract or other local scales. Mapping of Pb data is a key pillar underlying this goal and an initial step was undertaken to summarize existing Pb exposure and risk indices, environmental Pb indicators, and publicly available BLL data [14]. Challenges in data availability have inhibited further scientific progress in developing consistent and validated approaches for identifying high Pb exposure locations [14]. To address this need, Xue et al. [15] published a generalizable methodology for using individual-level BLL data and Pb exposure indices to evaluate the predictability of the latter for detecting true hotspots of EBLL at the census tract scale in the state of Michigan. Using Michigan’s extensive and robust BLL data, the results verified known hotspot locations and identified additional census tracts for follow-up [15].

This analysis further tests and demonstrates the generalizable mapping approach developed for MI [15] in another state with robust BLL data. Furthermore, it evaluates the consistency of findings across multiple periods and blood Pb reference values, including the current CDC reference value of 3.5 µg/dL. The focus was on Ohio, another midwestern state, where it has been shown that childhood blood Pb screening historically captured many of the EBLL cases that occurred [16]. According to a recent assessment by the Ohio Housing Finance Agency (OHFA), 67% of housing units in OH were built before 1980 and over 421,000 homes are an exposure risk to children for Pb-based paint [17]. The Ohio Department of Health (ODH) utilizes its BLL data for statistical modeling to identify high-risk zip codes for targeting blood Pb testing [18] and provides public access to summary statistics of BLL results, making it a good candidate for this analysis. The approach applied in this study augments ODH findings by providing finer scale resolution (i.e., census tract) that enables greater specificity in identifying neighborhoods where Pb exposure may be an issue for young children. In the current analysis, two different statistical methods were applied to children’s individual-level blood Pb measurements to determine the highest percentage EBLL census tracts and locations in OH. These “hotspots” were then compared with existing Pb exposure indices/models that are comprised of old housing and sociodemographic variables to determine how well these indices/models predict actual locations of EBLL.

## Materials and methods

### Data and materials

The OH blood Pb data analysis performed in this study was modeled after that done by Xue et al. [15]. In brief, BLL data were combined at the census tract scale and grouped by two- or three-year time periods to evaluate trends in children’s blood Pb exceedance rates (defined in Section “Statistical Methodology”) over time. Grouping census tract level BLL data in 3-year increments (i.e., 2005–2007, 2008–2010, 2011–2013, 2014–2016) made results more readily comparable to our initial work [15]. The remaining two-year increment (2017–2018) reflects the most recent available data in our dataset. We focused on examining the 2014–2016 results for consistency with prior work, since only 2 years of more recent data were available at the time of the analysis.

Once this study was reviewed and approved by institutional review boards at the ODH (2019-41) and the University of North Carolina at Chapel Hill, NC (16-2302), a data use agreement (DUA) between EPA and ODH was established. Through the Ohio Public Health Information Warehouse, we obtained individual children’s BLL records (ages <6 years) for 2005–2018 that included over 2.3 million (2,348,680) data points.

Using ArcGIS (version 10.6.1; ESRI Inc.), geocoding for this data was performed using the EPA Office of Environmental Information (OEI) Navteq USA Geocode Service (via the ESRI ArcGIS desktop software suite). To ensure function consistency and reliability, address matching was automated using service defaults and manual spot checks were conducted. Data were checked by an independent reviewer for quality assurance and quality control purposes (QA/QC). 2,115,827 data points were matched by point address; 188,514 matched by street address; and 7114 by street name. This resulted in 2,311,455 geocoded data points to include in the analysis with a 98.4% success rate (27 data points were later removed as they were geocoded to a location outside of Ohio resulting in a new total of 2,311,428). BLL records that could not be geocoded to a physical address were removed from the analysis (i.e., 37,225 records).

ODH provided census tract identification numbers (IDs) associated with their childhood BLL surveillance data. For QA/QC purposes, we performed an identity geoprocessing function in ArcGIS using the U.S. Census Bureau 2012 census tract polygon file to regenerate the IDs for the geocoded points possessing a point address, street address, and street name match type (the 2012 census tract file was selected as it represents an approximate middle year for the 2005–2018 BLL dataset). This process identified 98 duplicates for a total of 2,311,526 data points (2,311,428 of which were unique). Duplication occurred from points that overlapped census tract polygon boundaries, which was expected given that datasets were developed with different geographic datums and resolutions. We elected to retain duplicates and use the census tract IDs ODH provided as they were an almost identical match with our QA/QC findings.

ODH’s BLL testing requirements mandate that if a capillary sample result is ≥5 µg/dL, then it must be confirmed by venous draw. For this analysis, if multiple blood Pb tests were obtained for the same child in a given year, we randomly selected one measurement regardless of whether it was venous or capillary. As our objective was to obtain a representative sample of the population, we did not want to bias results high by selecting the confirmatory venous sample. Furthermore, our sample size would have been substantially reduced if we did not include capillary measurements, significantly reducing the ability to identify EBLL hotspots. Prior sensitivity analysis showed no appreciable difference in results based on selected measurements, likely due to the millions of data points included in the analysis [15]. There were 2,298,065 blood Pb measurements included in the final geospatial analysis, comprised of 1,305,106 capillary samples (57%), 882,819 venous samples (38%) and 110,140 unknown (5%). For blood Pb samples obtained from children residing in Canton during the years 2005–2013, the testing laboratories reported BLL at the detection limit of 5 µg/dL for any samples below this level, resulting in more than 50% of the capillary sample results reported exactly at 5 µg/dL [19]. Thus, Canton-affiliated census tracts were excluded from the analysis for these years at blood Pb reference levels of 3.5 and 5 µg/dL. Besides Canton, blood Pb laboratory analysis results below the limit of detection were not manipulated in the analysis.

### Statistical methodology

Robustness of the data set was determined based on blood Pb sample quantity [15]. Comparing two different EBLL metrics based on number of children tested and number of children living in each census tract enabled an evaluation of “representativeness” (Eqs. 1 and 2), whereas a high correlation between the rates demonstrated proportionality [15]. Once it was determined that the OH blood Pb data were robust and representative, we proceeded with identifying census tracts with EBLL using the exceedance rate.

The exceedance rate (as a percent) was the primary calculation used in the analysis and is defined per census tract as:

1 $$Exceedance\,rate( \% EBLL)=\frac{number\,of\,children\,tested\,with\,EBLL}{number\,of\,children\,tested\,in\,census\,tract\,}\times 100$$

The population rate (as a percent) is defined per census tract as:

2 $$Population\,{{{{{\rm{rate}}}}}}( \% EBLL)=\frac{number\,of\,children\,tested\,with\,EBLL}{number\,of\,children\,in\,census\,tract\,}\times 100$$

The exceedance rate calculation was used for producing geospatial results; the population rate was only used as a comparator with exceedance rate to characterize the robustness and representativeness of the blood Pb surveillance data.

Exclusion criteria for the analysis were: <50 children aged 0 to <6 years living in the census tract with a blood Pb test; <10 children aged 0 to <6 years tested in the census tract in a given year; and less than half of the years assessed not containing blood Pb data for the census tract.

Three different blood Pb reference values were used as thresholds for EBLL in the calculations: 10, 5, and 3.5 µg/dL. These values were selected to represent changes over time to the U.S. CDC blood Pb “level of concern” which was 10 µg/dL until 2012, when it was lowered to 5 µg/dL [8]. In 2021, CDC lowered the blood Pb reference value to 3.5 µg/dL to reflect the most recent data derived from NHANES [8]. As the calculations for exceedance rate and population rate were based on number of children with EBLL above the blood Pb reference value, there was no need to censor measurement data at or below the limit of detection (with the exception of Canton, as described previously).

### Geospatial analyses

Hotspots are defined in this analysis as geographic locations with higher prevalence of children’s Pb exposures, based on EBLL using two statistical methods: top 20th (80th–100th) percentile and Getis-Ord Gi* geospatial analysis [15]. The top 20th percentile method identified individual census tracts with the uppermost BLL (i.e., 80th–100th percentile). The Getis-Ord Gi* method [20] was executed using the ArcGIS Hot Spot Analysis tool (ESRI) set to a 95% confidence interval, which identified statistically significant hotspots of autocorrelated locations based on geospatial patterns of exceedance rates (%EBLL). “Reference locations” are defined as the highest population city (according to the 2015 Census Places file) near or containing a top 20th percentile census tract or more than one geospatial hotspot.

Further analyses were conducted based on Xue et al. [15] to classify census tracts as rural or urban to better inform where EBLL were occurring across the state. Census data [21] on rural and urban housing and population by age (from Summary File 1 H2 and P14, respectively) were joined to the results derived from both hotspot methodologies. Any census tract with >50% urban housing was designated as “urban” and any census tract with >50% rural housing was designated as “rural.” Briefly, the criteria for designating an area as urban was that it must be comprised of a densely settled core of census tracts and/or census blocks that encompass at least 2500 people; otherwise the area is considered rural^1^. Ratios between urban and rural census tracts identified as having hotspots and not having hotspots were calculated, along with total population aged 0 to <6 years old.

The exceedance rate Getis-Ord Gi* hotspot results were compared with environmental justice (EJ) variables at the census tract scale, following the methodology in Xue et al. [15]. Using EJSCREEN 2017 [22] and the 2011–2015 ACS [23], population variables (less than high school education, total minority population, low income, and non-Hispanic African American) were extracted, mapped, and spatially joined with the OH Getis-Ord Gi* results. Comparisons between hotspots and non-hotspots by both population number and percentage were completed and ratios derived.

Two publicly available indices, U.S. EPA’s EJScreen Pb Paint EJ Index and U.S. Housing and Urban Development’s (HUD’s) Deteriorated Paint Index (DPI), were applied to OH to evaluate the potential for identified hotspot census tracts to be explained by housing and demographic variables. In addition, a regression model for predicting children’s EBLL from the peer-reviewed literature [24] was applied, after being slightly modified (i.e., using 2012–2016 ACS data) to better align with the years included in this analysis [15]. We also applied a regression model for predicting the census tract geometric mean BLL (in µg/dL units) among U.S. children aged 12–35 months [24]. Trained on children’s BLL surveillance records from the State of Michigan Department of Community Health, years 1999–2009, the model includes coefficients for sample type (capillary or venous), child’s age in months, sampling year, and three census variables (percent of population below poverty line, percent pre-1960 housing, percent of population that is non-hispanic black). Our predictions were made for 24-month-old child, venous blood, and used 2012–2016 ACS data to better align with the years predicted in this analysis [15].

The spatial scale for these three indices/models were at the census tract to foster comparisons with the observed BLL data. Further motivation for this analysis was to assess if the use of existing indices/models may appropriately serve as surrogates in the absence of blood Pb measurements, particularly for states where universal blood Pb testing does not occur.

Cohen’s kappa statistic was calculated to compare hotspot results within different time periods; blood Pb reference values; spatial analysis approaches; and Pb indices/models for identifying potential hotspot locations. Interpretation of the kappa score is as follows: 0.41–0.6 indicates moderate agreement; 0.61-0.8 indicates substantial agreement; and 0.81–0.99 indicates near perfect agreement [25]. Visual inspection was also employed to characterize similarities and differences between OH census tracts across the various comparators.

Geospatial mapping and Getis-Ord Gi* hotspot analyses were done using ArcGIS version 10.6.1 and ArcGIS Pro version 2.8 (ESRI) and traditional statistics were performed using SAS version 9.4 (SAS Institute, Inc.).

## Results

**Fig. 1:**
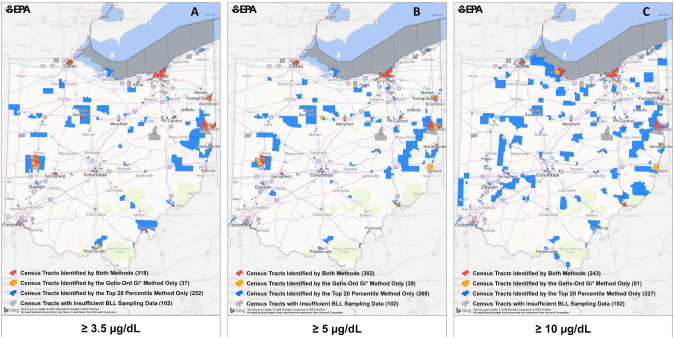
Comparison of hotspots for different blood Pb reference values. Individual panels represent different blood Pb reference values (**A** ≥3.5 µg/dL; **B** ≥5 µg/dL; **C** ≥10 µg/dL) hotspots identified using Getis-Ord Gi* geospatial analysis and top 20th percentile EBLL for 2014–2016 (children aged 0 to <6 years old). Colored census tracts represent hotspots (red = census tracts identified by both methods; orange = census tracts identified by Getis-Ord Gi* method only; blue = census tracts identified by top 20th percentile method only), whereas grey shading denotes census tracts excluded from the analysis due to insufficient BLL sampling data.

Figure 1 shows which census tract hotspots were identified for 2014–2016 using Getis-Ord Gi* and top 20th percentile for the various blood Pb reference values. There were 102 census tracts that were excluded in the analysis based on our inclusion criteria. The results are robust across different blood Pb reference values, with the most sizable hotspots identified at 3.5 µg/dL, also identified at 5 and 10 µg/dL (i.e., Cleveland, Toledo, East Liverpool). Similar findings were observed for the number of census tracts with percentage of EBLL ≥ 3.5 and ≥5 µg/dL using both Getis-Ord Gi* and top 20th percentile methodologies, with kappa scores all above 0.8 (Table S-1 and S-2). The kappa score was higher when the Getis-Ord Gi* methodology was applied compared to the top 20th percentile because this metric considers nearby census tracts to identify a hotspot. There was a slightly stronger agreement for the years 2014–2016 compared to 2017–2018, likely due to more years and census tracts being included in the former period. The agreement is not as strong for comparisons between 3.5 or 5 µg/dL and 10 µg/dL, albeit there is near perfect agreement for Getis-Ord Gi* when comparing 5 and 10 µg/dL for both periods. Given the near perfect agreement of identified census tracts between 3.5 and 5 µg/dL, we focused our results on the current CDC blood Pb reference value of 3.5 µg/dL.

Looking across the entire period evaluated (2005–2018), the number of census tracts with the highest EBLL have decreased over time (Table 1; Fig. S-1). In the earliest years, over 85% of census tracts had percentage of EBLL greater than 10% when using 3.5 µg/dL as the blood Pb reference value, which is over 23% of children statewide. Large reductions in BLL are observed when moving from 2008–2010 to 2011–2013. For the most recent years (2017–2018), only four census tracts are in the uppermost EBLL category and the statewide percentage of EBLL drops to 6.7%. The time trend shows the exceedance rate for all census tracts decreasing, with a few showing continued challenges with addressing children’s exposure to Pb. There were high correlation coefficients (>0.75) between exceedance rates (EBLL) and population rates using 3.5 µg/dL as the threshold when the Getis-Ord Gi* hotspot methodology was applied for all time periods (data not shown).

**Table 1:** Time series of the number of OH census tracts by percentage of children (0 to <6 years old) with elevated blood lead levels (EBLL) using 3.5 µg/dL as blood lead reference value.

	2005–2007	2008–2010	2011–2013	2014–2016	2017–2018
EBLL (%)	Number of Census Tracts				
0–5	95	328	1029	1385	1586
>5–10	289	759	862	777	677
>10–20	978	981	512	436	374
>20–40	956	490	292	231	182
>40–70	354	156	45	21	4
Total	2672	2714	2750	2850	2823
% Census Tracts EBLL > 10%	86	60	31	24	20
Statewide EBLL (Avg. % of children)^**a**^	23	15	9.6	8.3	6.7

^a^Calculated as number of children with BLL ≥ 3.5 µg/dL in OH, divided by number of children tested in OH.

As expected, the Getis-Ord Gi* analysis follows the same pattern, with fewer hotspots identified as time progresses (Fig. S-2). Major OH cities contain hotspots for the entire time series, although the sizes of the hotspots decrease over time (Fig. S-1). There was near perfect agreement when comparing the hotspots identified in 2014–2016 to 2017–2018 (kappa statistic = 0.84; Table S-3). The kappa statistic was a bit lower between 2014–2016 and 2017–2018 (0.69) using the top 20th percentile (Table S-4), which is largely attributable to the independent manner that the metric is derived. Unlike the Getis-Ord Gi* hotspot analysis, when using 3.5 µg/dL as the EBLL threshold for the top 20th percentile, the number of census tracts does not appear to decrease appreciably over time and shifts from urban to more rural locations (Fig. S-3).

When evaluating the Getis-Ord GI* hotspots for 2014–2016 and their associated reference locations (highest population city near or containing more than one geospatial hotspot), 355 census tracts (of 2850) were identified across 17 locations. Many of the census tracts are in the most populated cites, which include Cleveland (183) and Toledo (47) (Table 2). The highest percentage of EBLL was observed for Bellaire at 35%, which contained two census tracts in its hotspot with 214 children aged 0 to <6 years old living in this area. In addition, hotspots in Cleveland, Steubenville, Toledo, East Liverpool, Alliance, Mansfield and Springfield reference locations all had EBLL rates above 20%.

**Table 2:** List of Getis-Ord Gi* hotspot reference locations for 2014–2016 using 3.5 µg/dL as blood lead reference value (children 0 to <6 years old).

Reference Location^a^	Number of Census Tracts	EBLL (%)	Total Population 0 to < 6 Years Old
Alliance	4	23.1	1324
Bellaire	2	34.7	214
Canton	10	18.4	3274
Cincinnati	20	17.5	4604
Cleveland	183	25.1	33912
Columbus	11	19.0	4338
Dayton	3	14.5	650
East Liverpool	9	23.9	2161
Elyria	4	12.2	1019
Mansfield	5	22.9	1356
Portsmouth	4	19.4	957
Springfield	11	21.0	2758
Steubenville	4	25.1	756
Toledo	47	24.6	11823
Troy	8	19.5	3483
Upper Sandusky	2	17.1	520
Youngstown	24	19.9	4057

^a^A “Reference Location” is defined here as the highest population city (according to the 2015 Census Places file) near or containing more than one Getis-Ord Gi* hotspot census tract.

The U.S. Census Bureau definitions of urban census tracts included 353 of the hotspots, with only two being considered rural (Table S-5). When using the population of children aged 0 to <6 years old in these hotspots, many more children reside in urban than rural areas (over 77,000 and 541, respectively), providing strong evidence that the greatest areas of potential Pb exposure are in cities. The pattern holds when using the top 20th percentile metric, albeit there are many more children residing in rural census tracts with this methodology (Table S-5).

Further examination of these Getis-Ord GI* hotspots (containing 866,056 people) broken down by EJ-related variables provides information on how the population compares with non-hotspot census tracts (10,472,274 people) in the state (Table 3). There are over four times as many non-Hispanic African Americans living in OH census tracts identified as hotspots versus non-hotspots. This is consistent with the hotspot/non-hotspot ratio observed for total minority population which is over three times greater for hotspots. Less than high-school education and low income are also important EJ indicators of potential Pb exposure (Table 3).

**Table 3:** Comparison of environmental justice demographic information between EBLL non-hotspots and hotspots identified using Getis-Ord Gi* geospatial analysis and 3.5 µg/dL as blood lead reference value for 2014–2016 (children 0 to <6 years old).

	Non-Hotspot		Hotspot		Hotspot:Non-Hotspot Ratio of Percentages
	Population (10,472,274)	Percent	Population (866,056)	Percent	
Non-Hispanic African American^a^	1,005,328	9.6%	352,621	41%	4.2
Total Minority Population^b^	1,765,166	17%	463,627	54%	3.2
Less than High School Education^b^	713,348	6.8%	115,782	13%	2.0
Low Income^b,c^	3,244,343	31%	515,740	60%	1.9

^a^Data obtained from 2011–2015 ACS [23].

^b^Data obtained from EJSCREEN 2017 [22].

^c^Low income is defined as income less than two times the federal poverty level.

**Fig. 2:**
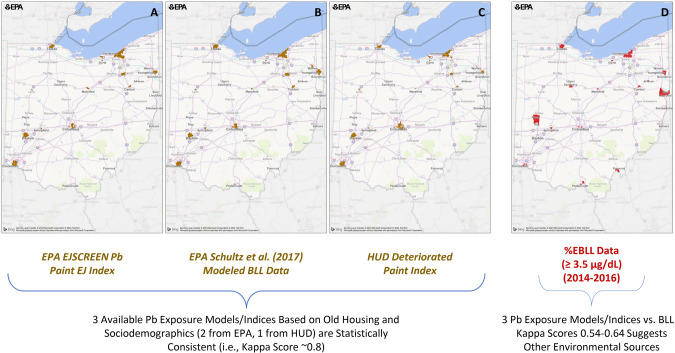
Census tracts identified using different Pb indices/model compared to EBLL data. The panels represent (**A**) EPA EJSCREEN Pb Paint EJ Index (2952 census tracts evaluated), (**B**) Schultz et al. regression model [24] (2952 census tracts evaluated), (**C**) HUD Deteriorated Paint Index (2372 census tracts evaluated), and (**D**) EBLL Getis-Ord Gi* geospatial analysis using a blood Pb reference value of 3.5 µg/dL for 2014–2016 (children aged 0 to <6 years old; 2850 census tracts evaluated). Legend note: brown = census tracts identified by index or model using Getis-Ord Gi* analysis; red = EBLL census tracts identified using Getis-Ord Gi* analysis.

There is moderate agreement across the three Pb indices/models (i.e., EPA EJSCREEN, Schultz et al. [24], and HUD DPI) evaluated for identifying census tracts of potential high Pb exposure using Getis-Ord Gi* EBLL (Fig. 2; Table S-6). There was consistency when each index/model was compared against the others (kappa statistic ≥0.75). This is not surprising, given that the variables that derive these indices/models are similar. The variable common to all indices are housing age, albeit HUD DPI is based on predicted percent of pre-1980 housing at risk of containing deteriorated paint based on data from the American Housing Survey (AHS) and American Community Survey (ACS) [26]. EJSCREEN 2017 Pb Paint EJ Index and Schultz et al. [24] both include percent of pre-1960 homes, economic indicators (i.e., percent low income and percent below the poverty line, respectively), and minority populations (i.e., percent minority and percent non-Hispanic African American, respectively). EJSCREEN 2017 Pb Paint EJ Index and Schultz et al. [24] evaluated all 2952 OH census tracts, whereas the HUD DPI evaluated 2372 census tracts. When the Pb indices/models were compared with the Getis-Ord Gi* hotspot results, the agreement was not as strong (kappa statistic ranged from 0.54 to 0.64; Table S-6), although visual inspection (Fig. 2) shows many overlapping reference locations that include areas of Toledo, Cleveland, Columbus, Youngstown, Springfield, Canton and Cincinnati. The hotspots that do not align well with the Pb indices/model are Troy, East Liverpool, Upper Sandusky, Pomeroy and Alliance.

## Discussion

The results demonstrate that the OH children’s blood Pb surveillance program is robust and representative based on the calculated exceedance rate and population rate and that BLL data can be used in the generalizable approach initially published in Xue et al. [15] in other states or locations. The kappa statistic over time showed substantial to near perfect agreement, proving that visual observation translated to quantitative concordance. This is further supported by an analysis by Roberts et al. [16] who report a high ascertainment ratio (0.93) for OH that reflects a high likelihood that testing is comprehensively capturing those children with EBLL. Although the statistical modeling was conducted for 1999–2010 using 10 µg/dL for calculating prevalence [16], the ODH blood Pb screening guidance has been consistent, such that these findings should hold for the years included in the current analysis.

Comparing results across different EBLL thresholds facilitates evaluation of the ability to properly detect census tracts of potential Pb exposure. We demonstrated that the most recent blood Pb reference value (3.5 µg/dL) can be used with the derived BLL metrics and spatial analysis approaches to identify areas for targeting. The strong agreement between 5 and 3.5 µg/dL results for the years analyzed provides confidence that blood Pb surveillance programs will continue to identify areas containing children most at risk for Pb exposure. The limit of quantification per the ODH for capillary measurements is 3.3 µg/dL, so using a cutoff of 3.5 µg/dL should not result in misclassification. In addition, the EBLL rate utilizing percentages rather than absolute values was intentionally derived for the generalizable approach to avoid potential challenges in having to consider how to handle limits of detection at the lower end of the BLL distribution.

The observed decrease in EBLL over time in OH was also observed for MI [15]. As with MI, the biggest gains in lowered numbers of census tracts with EBLL in OH were from 2005–2010, with smaller improvements from 2011–2018. Similarly, there was a shift in the BLL distribution to the left, with fewer census tracts identified in the later years as being in the upper percentiles for EBLL. For 2014–2016 with a blood Pb threshold of 5 µg/dL, the percent of census tracts in OH with EBLL of >10% was 10.9% compared to 7.7% for MI and the statewide percentage being 4.5% compared to 4.1%, respectively. The 2017–2018 data for OH puts the number of census tracts with the percent of EBLL > 10% similar to MI at 8.6%. These data can be contrasted to the 2.5% national EBLL percentage that is used by CDC in their blood Pb reference value that was lowered from 5 to 3.5 µg/dL in 2021.

From 2007–2018, over 150,000 children aged 0 to <6 years of age were tested each year for blood Pb in OH [27]. Of those, 2.3 to 2.8% of children tested had a confirmed EBLL of 5 µg/dL or higher [28]. OH requires a confirmation venous draw for any capillary blood Pb result that is ≥5 µg/dL to identify individual children who require follow-up care. In our analysis, the statewide EBLL percentage was 3.5% for 2017–2018 when EBLL was defined as ≥5 µg/dL, which is higher than the 2.3 to 2.8% reported by ODH [28]. This likely reflects the inclusion of both capillary and venous blood Pb data in our methodology. This choice reflects our interest in examining individual BLL data for patterns at the community level, with the goal of providing valuable insights for targeting outreach or mitigation efforts at this broader scale.

In a study that evaluated findings of OH site investigations of children with reported BLL > 10 µg/dL, deteriorated interior paint, deteriorated external paint, dust and bare soil were most identified as hazards [29]. Of the 5% of cases where a bare soil sample was collected, Pb concentrations were often in excess of 400 ppm, with the greatest concentrations occurring closest to building structures where exterior paint and drip lines may be potential Pb sources. These assessments most often occurred in Mahoning County (that contains Youngstown) and Hamilton County (that contains Cincinnati). Interestingly, higher soil Pb concentrations did not necessarily correlate with EBLL where soil was the only detected hazard, indicating that other sources were likely contributing to those cases.

Many of the variables linked to hotspots in the current analysis are also considered as risk factors by ODH (i.e., home age, deteriorated paint, and income level), which provides confidence in the results. OH law requires blood Pb testing for children at ages 1 and 2 years under any of the following circumstances (1) child is on Medicaid; (2) child lives in a zip code distinguished by ODH; (3) child lives or regularly visits a home, child care facility or school built before 1950; (4) child lives or regularly visits a home, child care facility or school built before 1978 that has deteriorated paint; (5) child lives or regularly visits a home, child care facility or school built before 1978 that has recent ongoing or planed renovation/remodeling; (6) child has sibling or playmate that has EBLL; (7) child comes into contact with an adult who has a hobby involving Pb or works with Pb; and (8) child lives near an active or former Pb smelter, battery recycling plant or other industry known to generate airborne Pb dust [30].

The ODH targeted testing plan for Pb screening shows that housing built before 1950, population of non-Hispanic African Americans, population with a high school education, families whose income-to-poverty ratio was greater than two, and population under the age of 6 years were the most significant predictors of EBLL at the census tract scale [18]. This was consistent with earlier work to develop a Pb risk indicator at the census tract scale for OH using 1997 BLL data, which included housing built before 1950, residents of non-Hispanic African American ethnicity, residents with less than a high school diploma, and housing units that are renter-occupied in the final model [31]. Both populations of non-Hispanic African Americans and populations with less than a high school education were much more likely to reside in an identified hotspot (by four or two times, respectively) in our analysis, providing further confirmation of prior findings. These EJ-related indicators appear to be stable over time, with BLL data from the latest analysis highlighting the same variables as those identified over 20 years ago.

LSLs in drinking water distribution systems can be a potential source of household Pb exposure and contributor to children’s EBLL. Although a comprehensive inventory of LSLs across the U.S. does not exist, estimates have been calculated based on individual community water system (CWS) surveys. The midwestern U.S. has the largest share of LSLs, representing over half of those in the entire U.S., with OH and Illinois estimated to have the greatest number of LSLs in the region [32]. Housing age has also been linked to LSL prevalence, with greater percentage of older housing (pre-1960) related to increased percentage of water systems with LSLs [32]. Thus, older housing as an identified risk factor in this region could reflect both presence of Pb-based paint and LSLs.

In the analysis for OH, more census tracts in rural areas away from major metropolitan areas were identified with the top 20th percentile approach than the Getis-Ord Gi* approach. This is consistent with the top 20th percentile results from MI, where 44% of the census tracts with EBLL did not overlap with the hotspots identified using Getis-Ord Gi* and generally were in more rural locations. There were 102 OH census tracts that were not included in the EBLL geospatial analysis, most of which were in urban areas (73 urban, 19 rural, 10 neither). While some rural census tracts were identified with our methodology, it is possible that children in these communities are not regularly screened for blood Pb, resulting in bias toward urban areas. Further evidence may be provided in the OH childhood blood Pb testing requirements [30] that specify high-risk zip codes where screening is mandatory; there are seven OH counties that do not have zip codes on this list (Adams, Fulton, Highland, Jackson, Paulding, Pike, and Union) and six of these are considered “rural”^2^ based on the 2020 Census^3^. Prior work from ODH demonstrated that both Carrol County and Morrow County (classified as rural by the U.S. Census Bureau^4^) had high observed probabilities and low predicted probabilities of BLL ≥ 5 µg/dL in logistic regression modeling that included a limited number of BLL samples from rural areas [18].

As shown by the urban/rural categorization, the geospatial hotspot approach is better at detecting urban communities of multiple census tracts where housing age is more homogenous. Older housing is found in greater quantities in urban areas of OH, with 89% of units build prior to 1980 and over 57% built before 1950 [17]. A study in Toledo, OH reported age of housing as a key indicator for children’s BLL > 5 µg/dL in the urban core but was not a strong indicator for more rural areas [33].

By visual inspection, the high BLL zip codes specified by ODH in their blood Pb screening criteria [30], contain nearly all the Pb hotspots identified using our geospatial approach, including the largest cities of Cleveland and Toledo. The mapped results from the Pb indices/models compared well with EBLL data using 3.5 µg/dL at the threshold for these two cities, as well as Youngstown, Mansfield, Springfield, Elyria, and Canton. Thus, old housing and demographics are good predictors of Pb exposure in these cities and in the absence of any measured blood Pb data, these tools could be used to target education or mitigation efforts.

In contrast, the reference locations of Troy and East Liverpool that collectively contain nearly 20 census tracts and over 5000 children aged 0 to <6 years were not identified in maps produced from the Pb indices/models. The zip codes containing Troy and East Liverpool are included as high risk by ODH that require blood Pb testing for young children who reside in these areas [30]. In these communities, sources of Pb exposure are not readily explained by housing age, race/ethnicity, or income variables in our analysis, suggesting that community wide environmental Pb sources or other risk factors may be contributing. One possibility is exposure from Pb ammunition, which has been shown to result in high soil Pb concentrations [34] and elevated EBLL [35], including at firing ranges. Take-home Pb dust adhered to shooters may result in a residential exposure pathway for children [34, 35] that would not be captured herein. It is noteworthy that ODH does include casting ammunition as a listed hobby for adults who are in frequent contact with children less than 6 years of age on their survey instrument for required blood Pb testing [30]. Non-environmental sources could also be contributing, particularly for refugees who settled in OH and were exposed to Pb prior to immigration [36]. While the existing Pb indices/models do relatively well to predict census tracts containing a few key Pb exposure determinants (largely in urban areas), there will be locations containing differing Pb risk factors that will prohibit identification. Without widespread investigation or a comprehensive screening program, it could be challenging to identify children in these communities who are at increased risk for blood Pb levels above CDC reference values.

The statistical agreement between the Pb indices/model and OH EBLL hotspot data in this study ranged between 0.54 and 0.64, with the Schultz et al. [24] model having the highest kappa value. Both EJ Screen Pb Paint EJ index and the HUD DPI had weaker agreement with the observed OH data (kappa = 0.58 and 0.54, respectively), demonstrating that the Schultz et al. [24] regression model derived with BLL measurements was a better predictor of hotspots despite being developed using data from other states. It is also noteworthy that the HUD DPI is a household-level predicted risk metric for homes that may contain large areas of peeling paint [14], and not intended specifically for identifying EBLL hotspots for young children. When the same comparison between the Pb indices/model was done for MI EBLL hotspots (albeit using 5 µg/dL for the blood Pb reference value), kappa values were quite similar (i.e., between 0.54 and 0.55) [15]. Possible explanations for the slightly higher kappa values for OH may be more census tracts being included for EJSCREEN and Schultz et al. [24] and the lower threshold for the blood Pb reference value. Regardless, the findings for OH and MI are consistent and provide evidence that many locations of potential Pb exposure can be identified using these approaches.

Our results suggest that the generalizable methodology previously developed [15] is helpful to inform further investigation and other actions in response to potential high-risk Pb exposures for children living in hotspots in OH. The successful application of the approach and the moderate agreement with public Pb indices (i.e., EPA EJ SCREEN Pb paint EJ Index and HUD DPI) provide additional confidence that these indices can be used in the absence of sufficient available blood Pb testing data to screen for potential Pb exposure risk [37]. This brings us closer to having a consistent approach (i.e., whole-of-government systematic roadmap or blueprint as described in [14]) that can be applied on the national scale or state-specific scale to target those areas where Pb continues to be problematic. However, census tracts affected by environmental sources that are not associated with housing age or demographics will likely not be identified by these indices.

Universal blood Pb testing can inform targeted actions to help children after they are exposed and identify places needing more attention; surrogate Pb indices can inform preventative efforts before exposure occurs. For states with robust and representative BLL data at the community scale, investigations to pinpoint Pb hazards and on-the-ground knowledge of contamination or other risk factors could provide insight into exposure drivers. Future work could enhance existing statistical methodologies by incorporating environmental sources to this approach that can be applied in states without extensive BLL data.

Inequities in EBLL prevalence persist, despite the progress in reducing sources of Pb exposure. Non-Hispanic African American, less than a high school education and income less than two times the federal poverty level are indicators of much greater risk of residing in an identified hotspot in both OH and MI. It is important to recognize that these overlapping characteristics are often found in environmental justice communities who experience disproportionate and cumulative exposures that may magnify the health impacts from Pb. OH has prioritized testing for Pb exposure, particularly for those children in high EBLL prevalence communities and of low income [38]. Continued and expanded screening for BLL is essential for mapping and targeting high Pb exposure risk locations and exposure disparities and for tracking progress as the U.S. strives to achieve primary and secondary prevention of Pb exposure [11].

### Supplementary information

Supplemental Information

Supplementary Figures 1,2 Data

## Data Availability

This manuscript was prepared using statewide blood lead data through the Ohio Public Health Information Warehouse under an approved Data Use Agreement with the Ohio Department of Health. The Department specifically disclaims responsibility for any analyses, interpretations, or conclusions from these data. Individual level data are not publicly available, as they were obtained through a Data Use Agreement. Census tract level data that comprise Figs. 1 and 2 are available as Supplementary Material.

## Footnotes

1 https://www.census.gov/programs-surveys/geography/guidance/geo-areas/urban-rural/2010-urban-rural.html

2 The percentage of population within rural blocks range from 64% to 100% rural

3 https://www2.census.gov/geo/docs/reference/ua/2020_UA_COUNTY.xlsx

4 https://www2.census.gov/geo/docs/reference/ua/2020_UA_COUNTY.xlsx
