# Supplementary material for: Identification of high lead exposure locations in Ohio at the census tract scale using a generalizable geospatial hotspot approach
Source: J Expo Sci Environ Epidemiol. 2024 Apr 4;34(4):718–26. doi: 10.1038/s41370-024-00666-x (PMC11303242; doi:10.1038/s41370-024-00666-x)
Supplement: Supplementary file 1 — Supplemental Information [file 41370_2024_666_MOESM1_ESM.docx]

**Supplemental Information for:**

Identification of High Lead Exposure Locations in Ohio at the Census Tract Scale using a Generalizable Geospatial Hotspot Approach

This file contains three figures and six tables showing visual and statistical comparisons between time periods for individual census tracts and the two different hotspot census tract identification methods (i.e., Getis-Ord Gi* and top 20^th^ percentile). Number of census tracts broken out by urban and rural categories for the two identification methods are also presented, as is the comparison between the different Pb indices/models with hotspot results.

Figure S-1. Time series of percent EBLL using 3.5 µg/dL for blood Pb reference value by census tract (children 0 to <6 years old). Note: For the years 2005-2013 in Canton, blood Pb sample results below the laboratory detection level were reported by the testing laboratory as 5 µg/dL; these data are grey in the maps below.


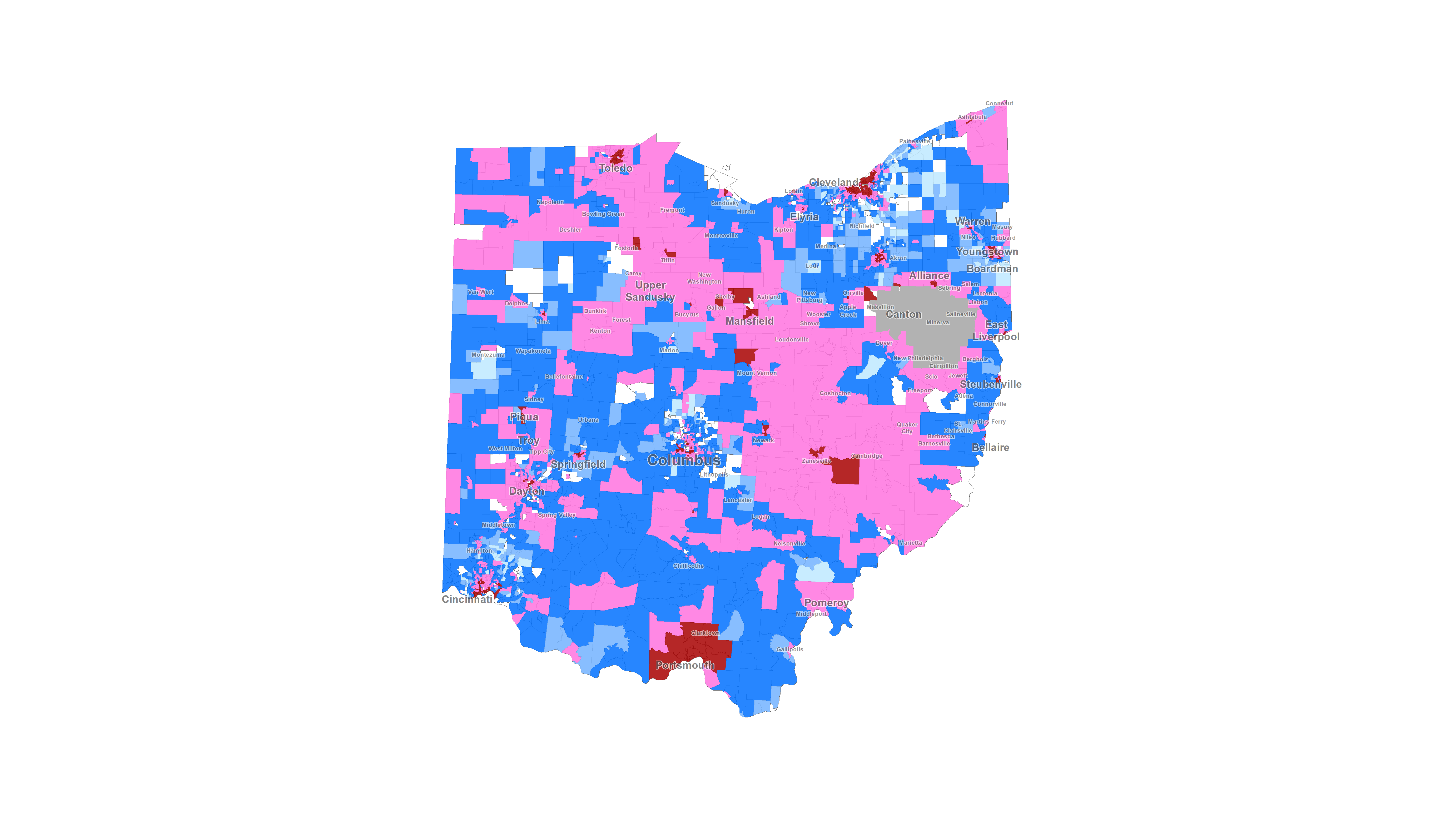

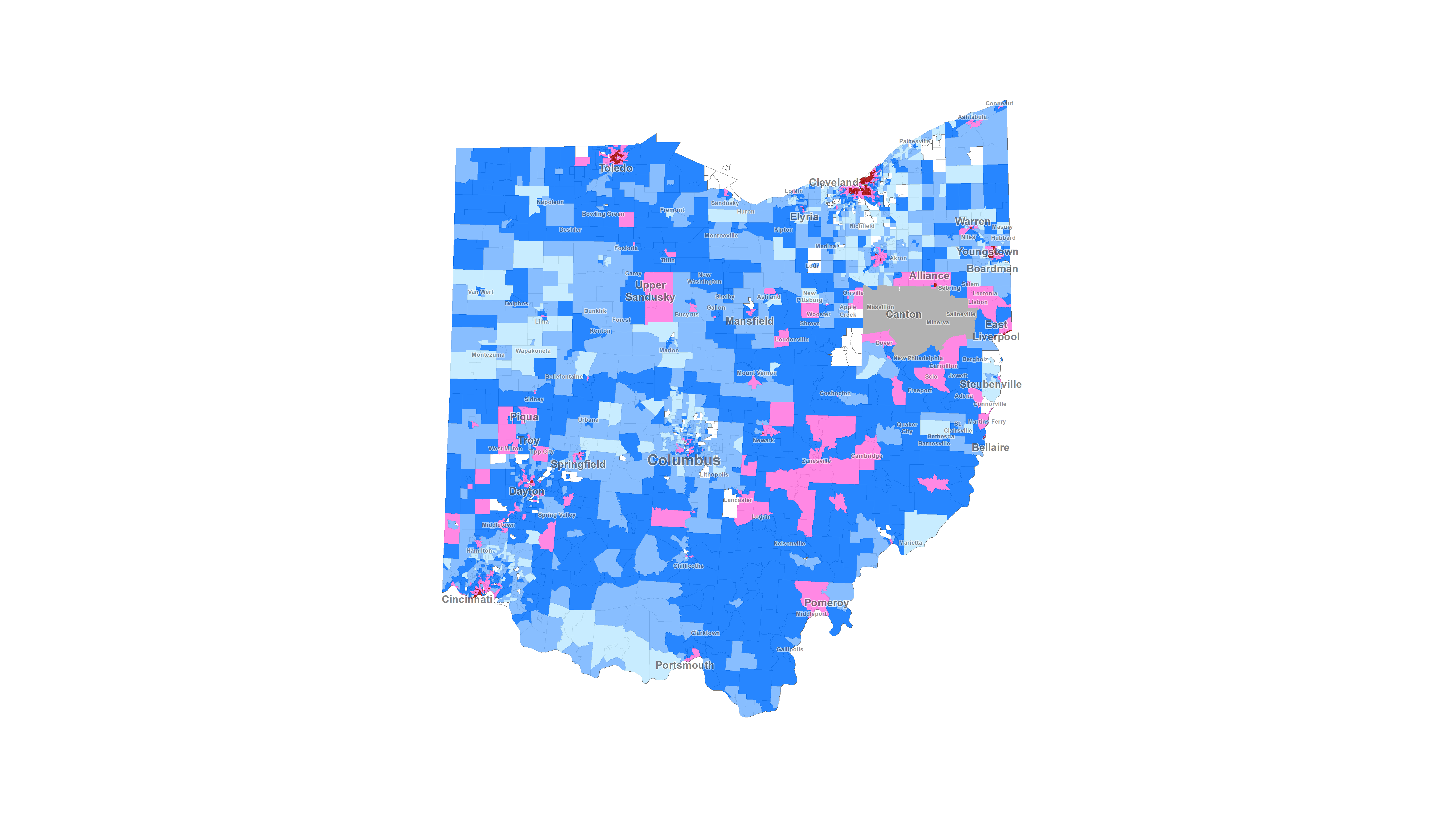

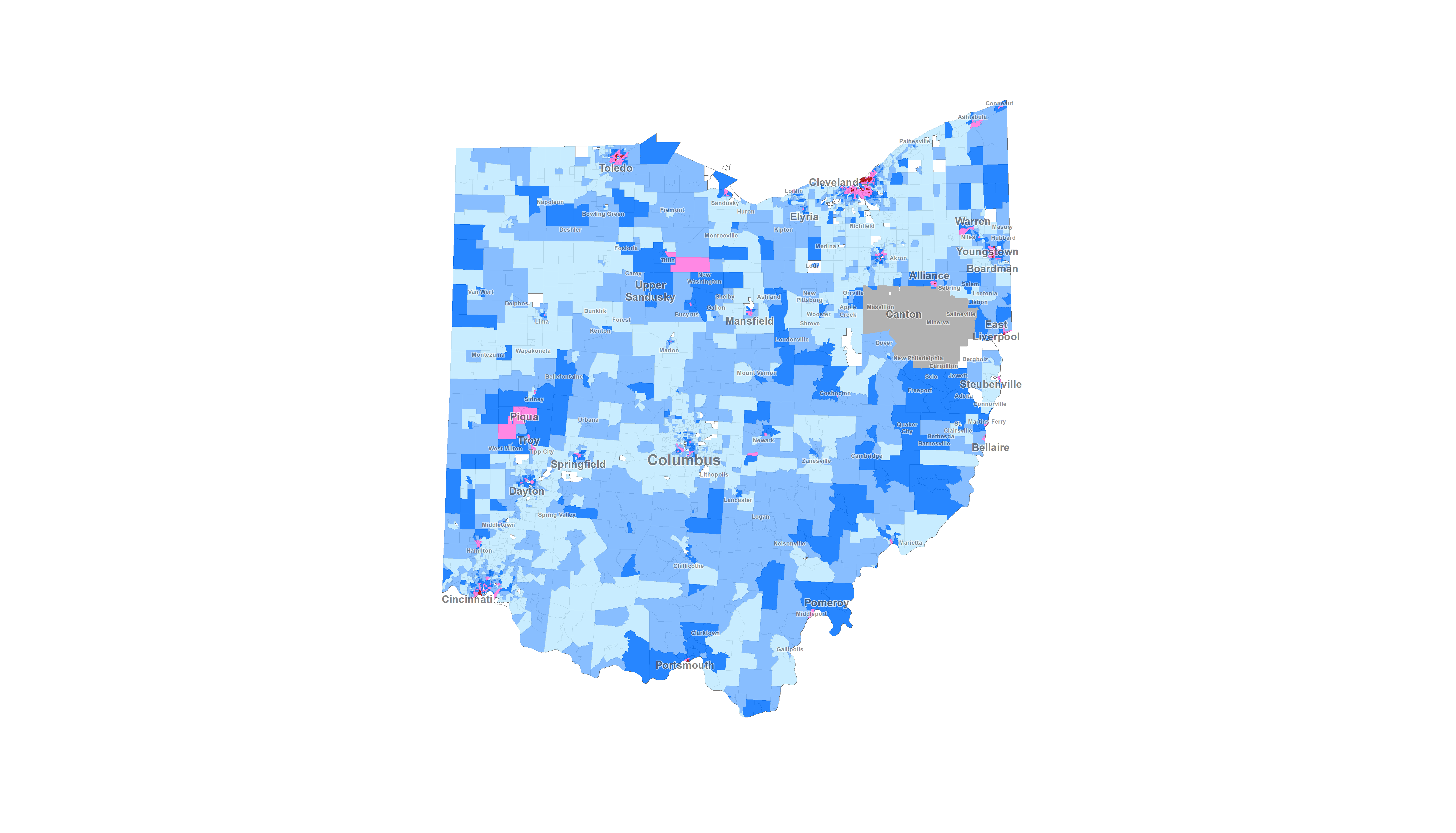


2011 - 2013

2005 - 2007

2008 - 2010

0 – 5%

> 5 – 10%

> 10 – 20%

> 20 – 40%

> 40 – 70%

Canton area


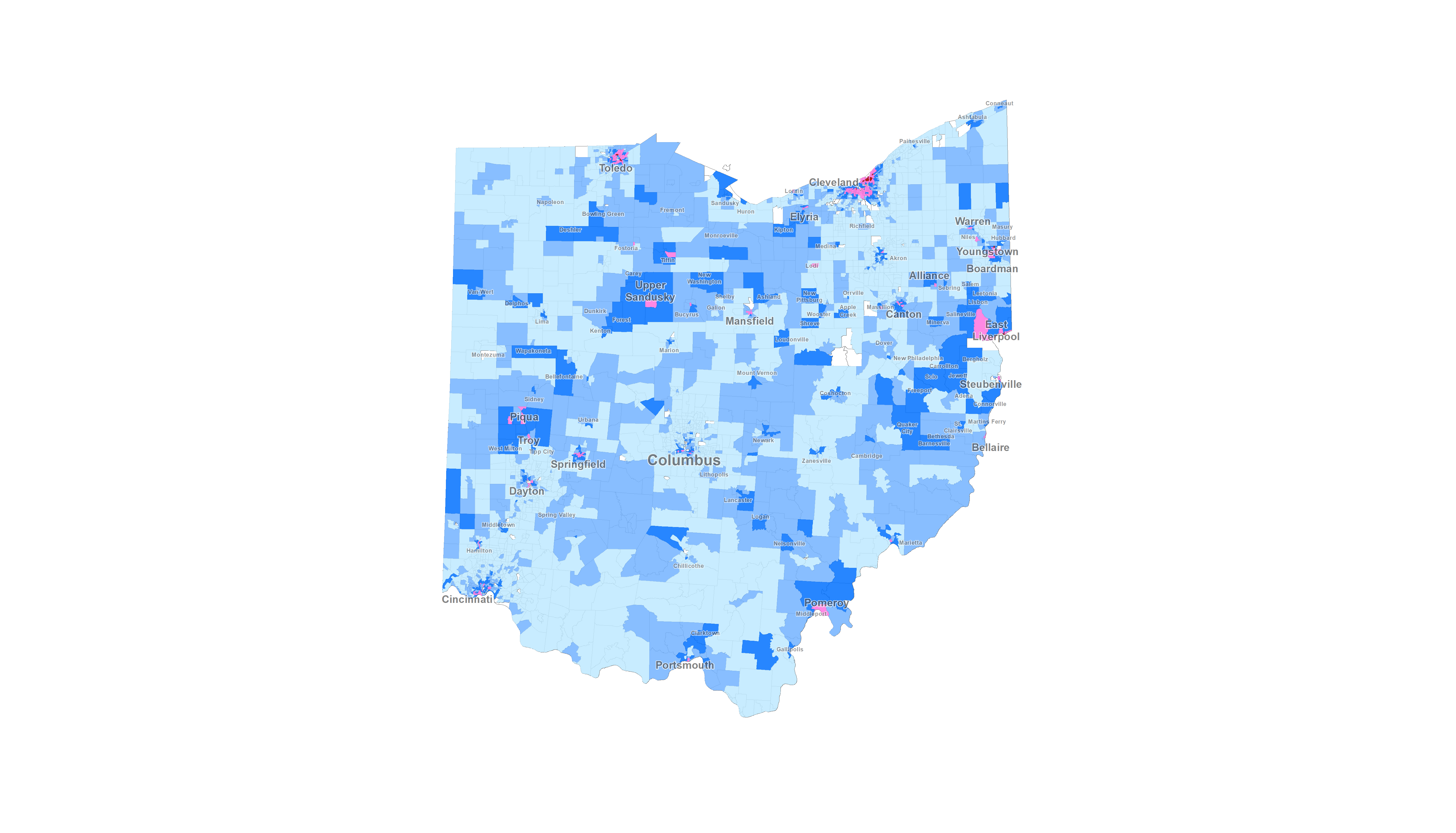

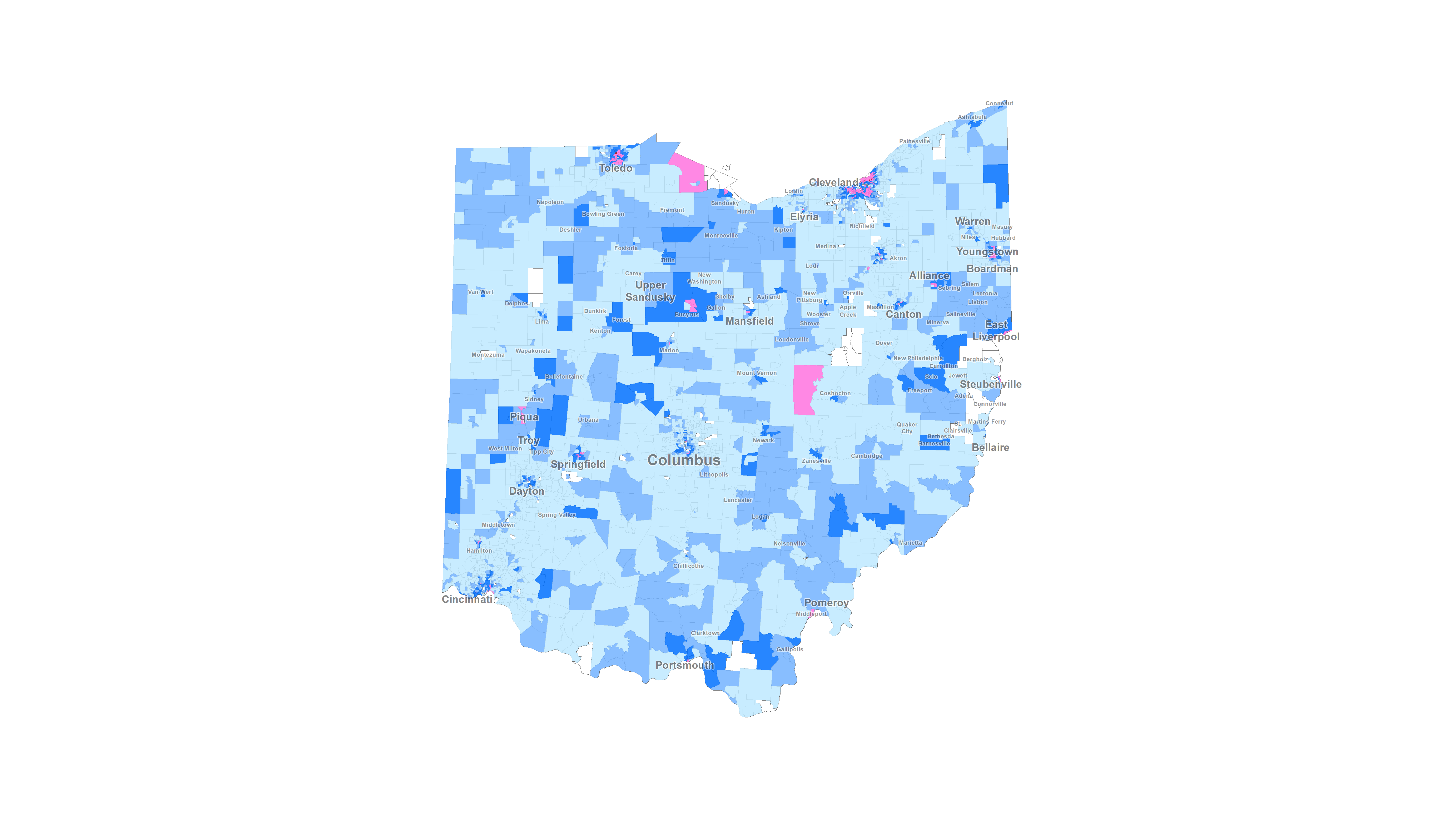


2017 - 2018

2014 - 2016

Figure S-2. Time series (A: 2005-2007; B: 2008-2010; C: 2011-2013; D: 2014-2016; E: 2017-2018) of Getis-Ord Gi* geospatial hotspots for EBLL using 3.5 µg/dL for blood Pb reference value (children 0 to <6 years old). Note: For the years 2005-2013 in Canton, blood Pb sample results below the laboratory detection level were reported by the testing laboratory as 5 µg/dL; these data are grey in the maps below.


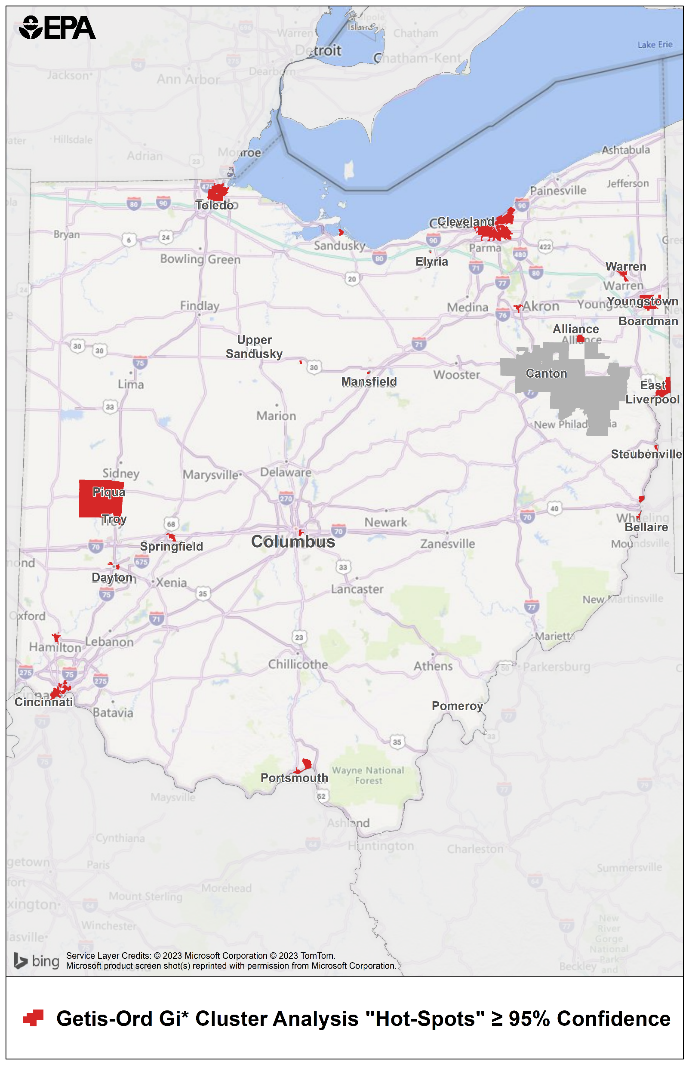

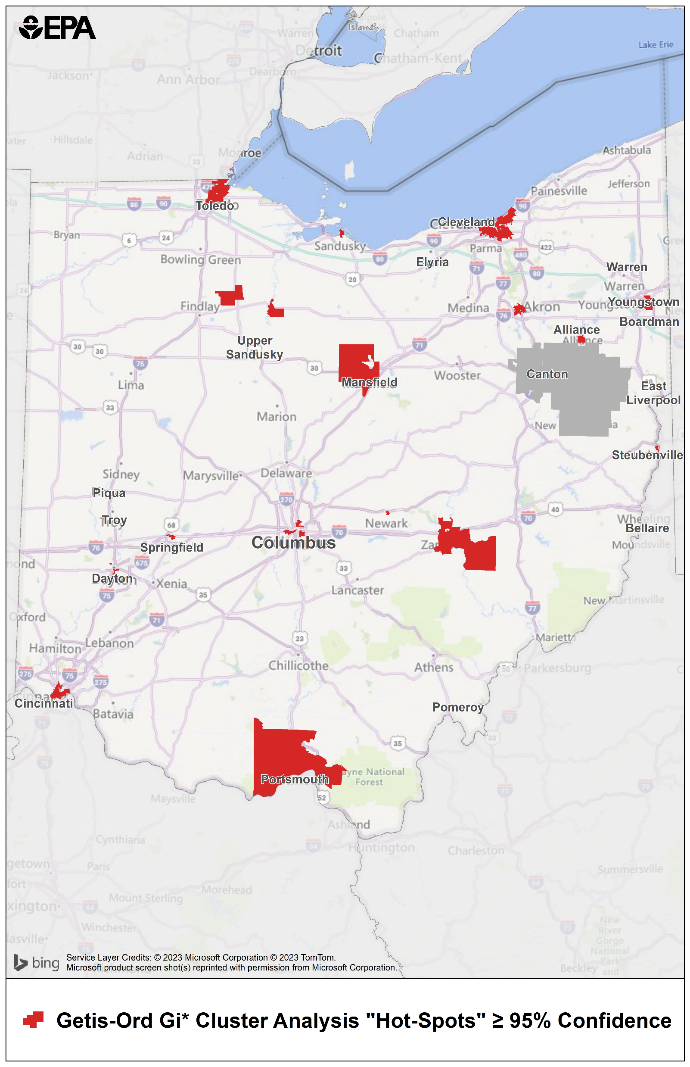

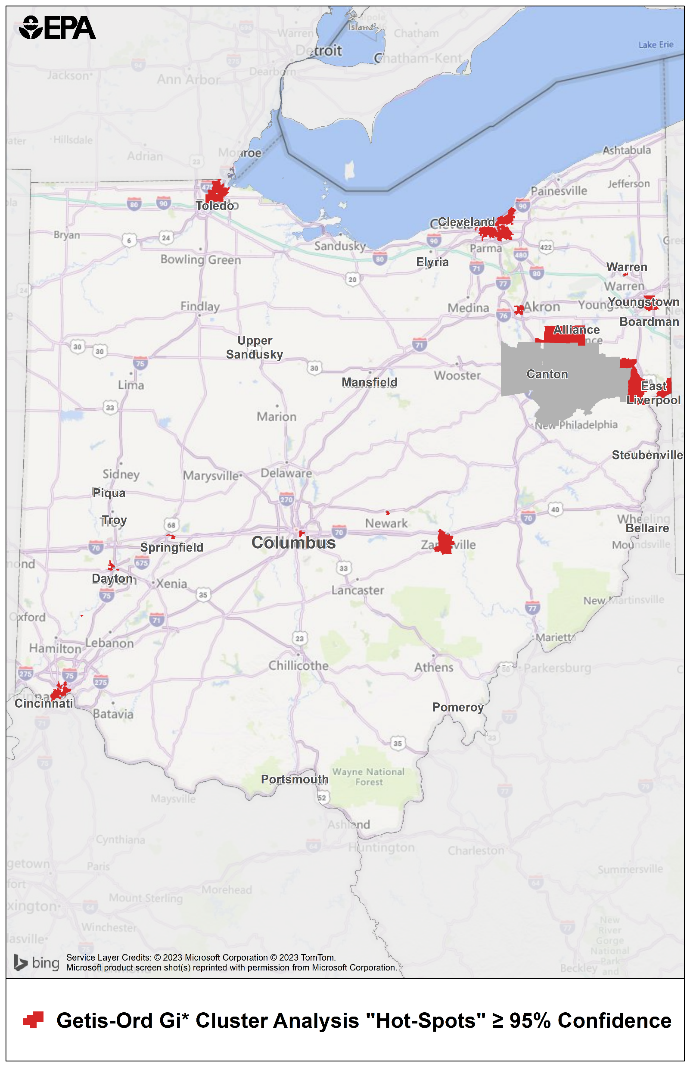


**A**

**B**

**C**


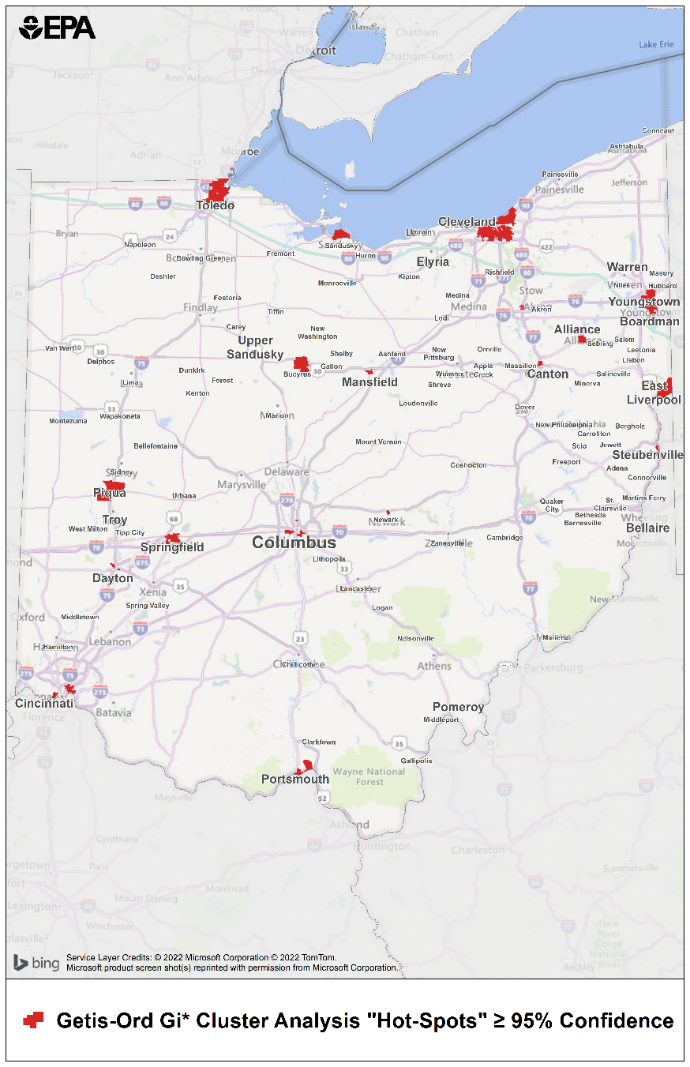

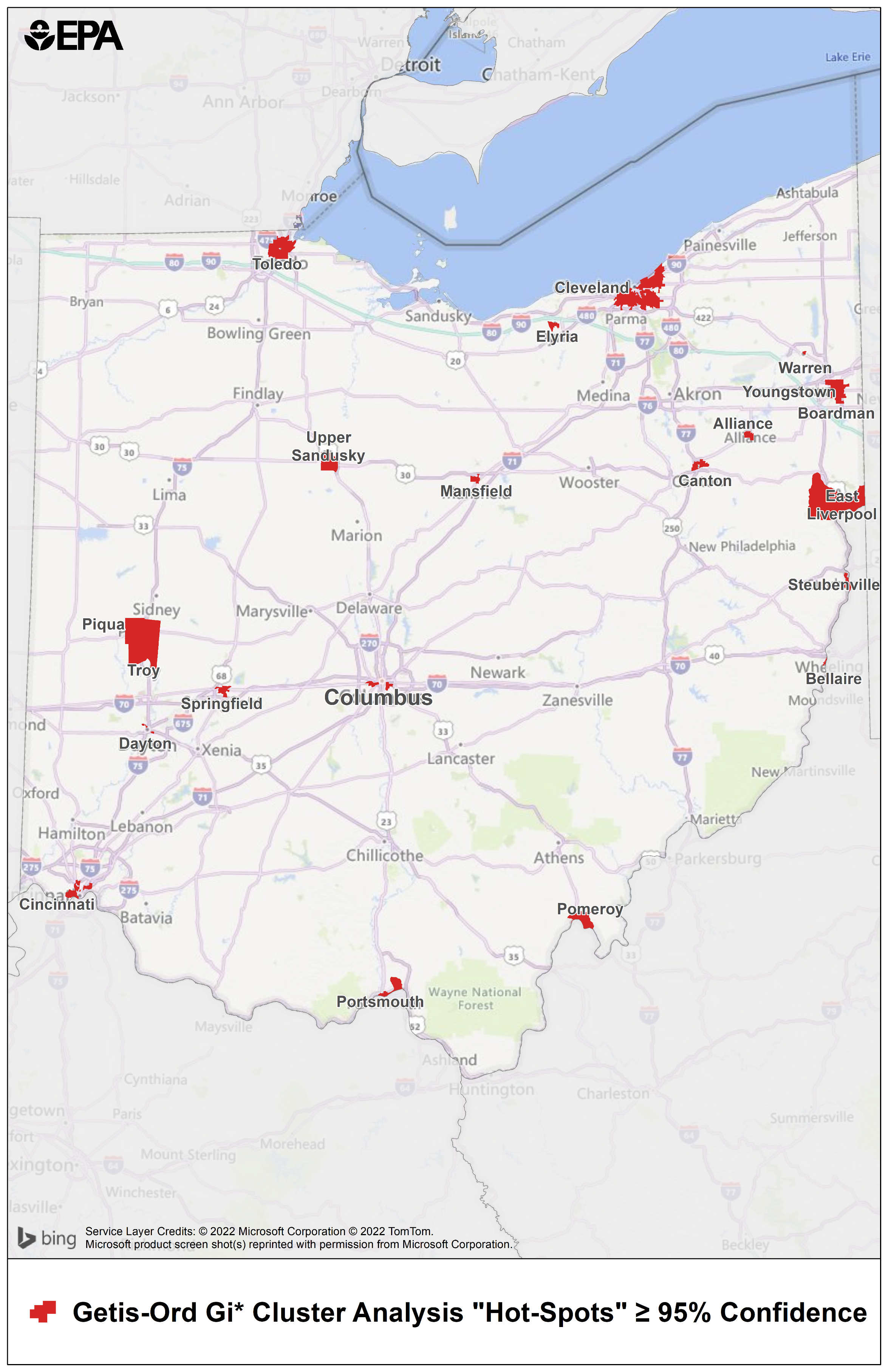


**E**

**D**

Figure S-3. Time series (A: 2005-2007; B: 2008-2010; C: 2011-2013; D: 2014-2016; E: 2017-2018) of top 20^th^ percentile census tracts for EBLL using 3.5 µg/dL as blood Pb reference value (children 0 to <6 years old). Note: For the years 2005-2013 in Canton, blood Pb sample results below the laboratory detection level were reported by the testing laboratory as 5 µg/dL; these data are grey in the maps below.


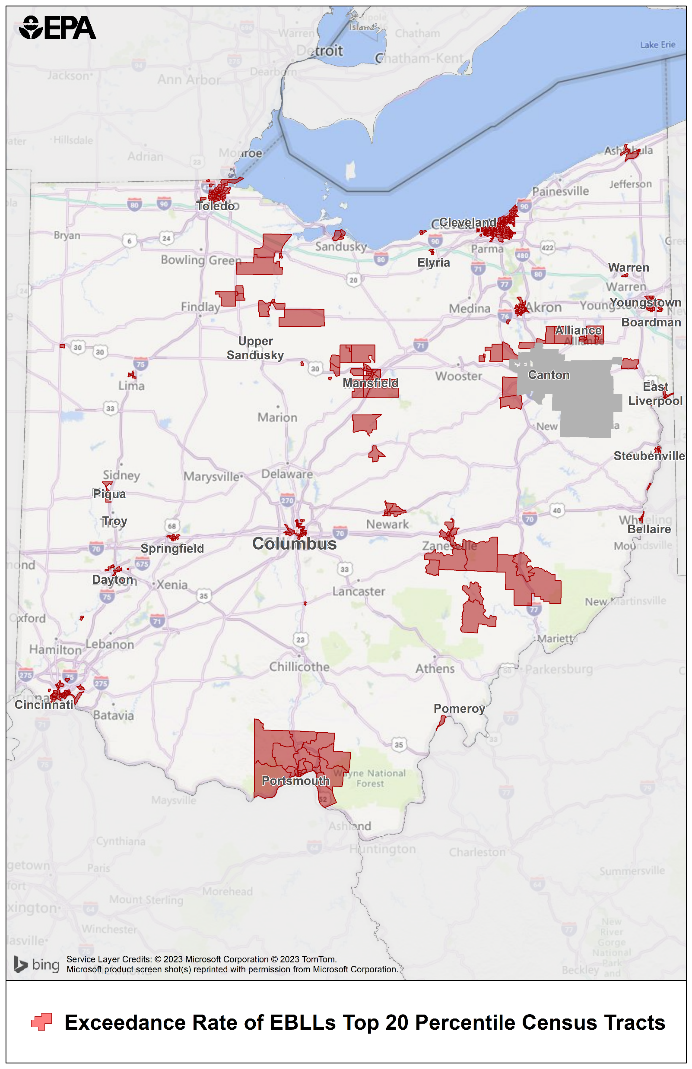

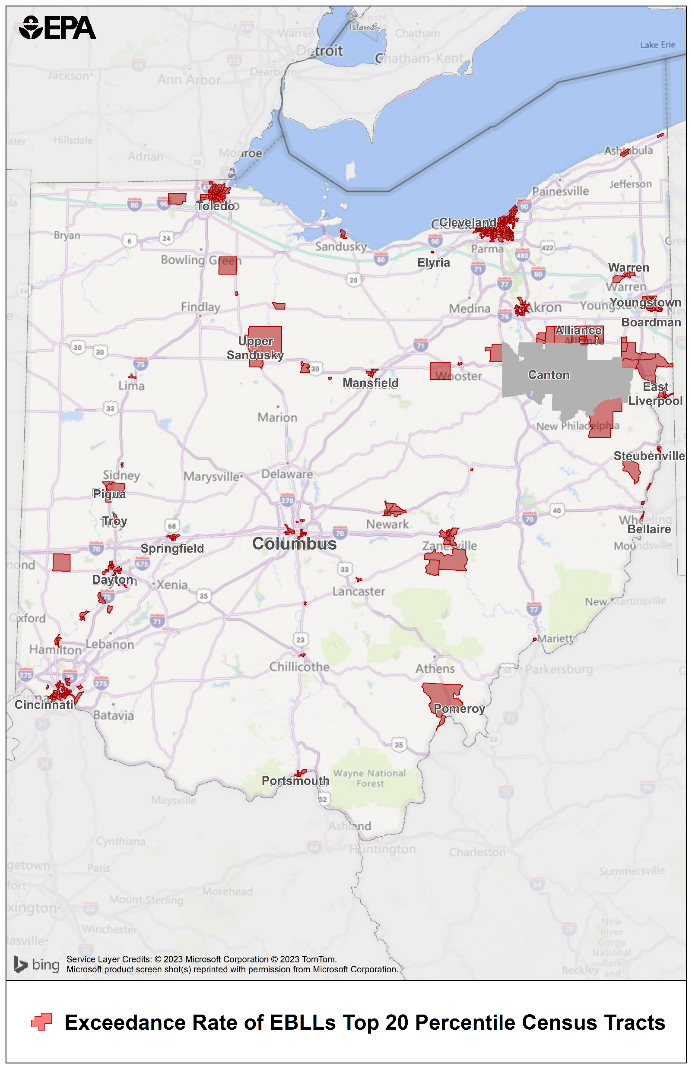

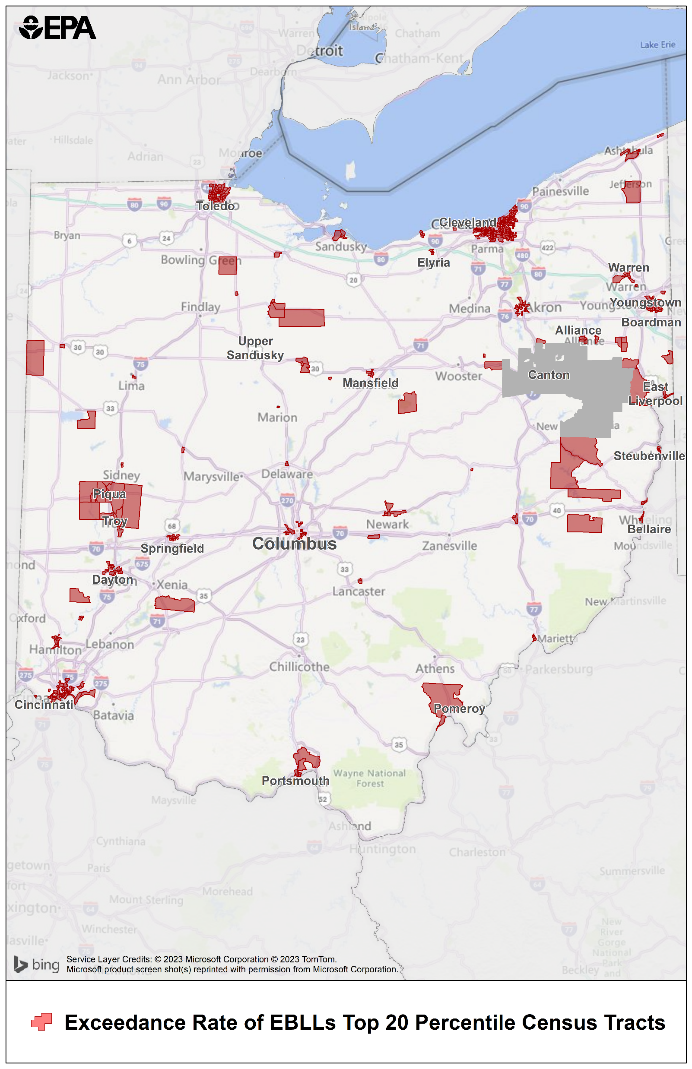


**A**

**C**

**B**


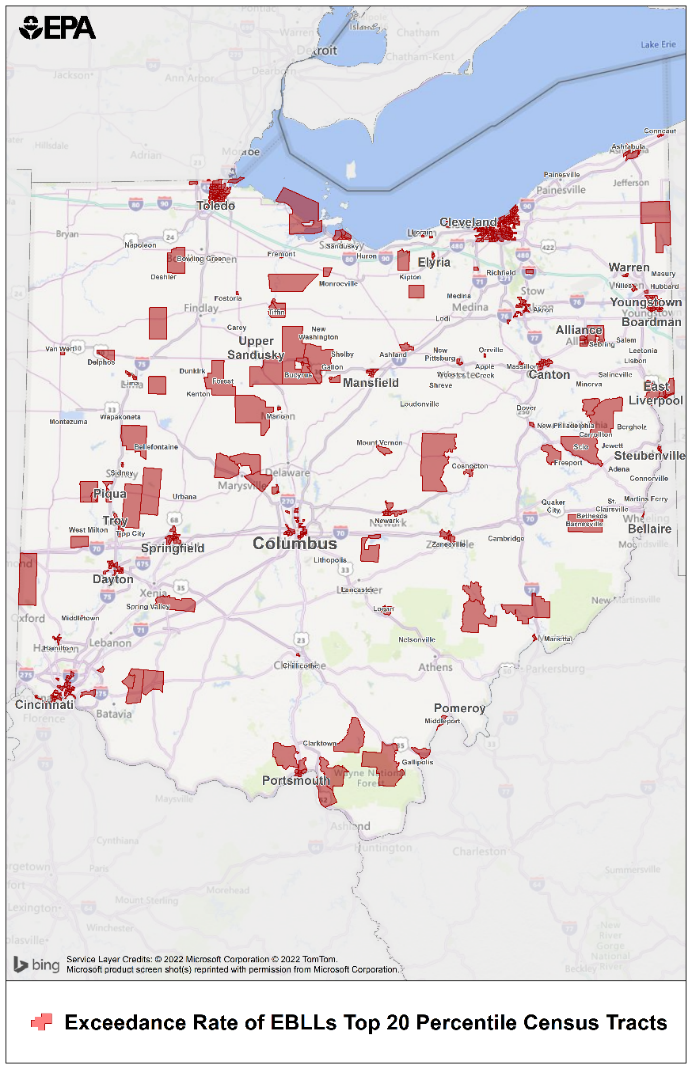

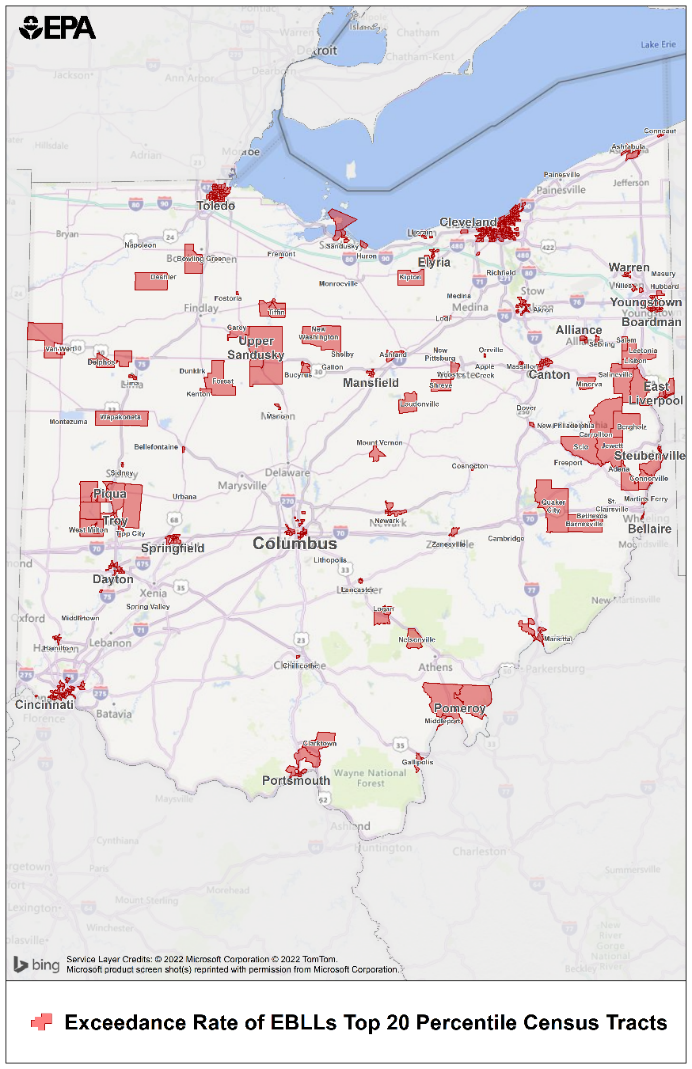


**D**

**E**

**Table S-1. Agreement between different blood Pb reference values for two time periods using the Getis-Ord Gi* method (children 0 to <6 years old)**

| **Blood Pb Reference Values** | **Period** | **N^a^** | **Kappa^b^** |
| --- | --- | --- | --- |
| **≥3.5 vs. ≥5 µg/dL** | 2017-2018 | 2823 | 0.92 |
|  | 2014-2016 | 2850 | 0.93 |
| **≥3.5 vs. ≥10 µg/dL** | 2017-2018 | 2823 | 0.79 |
|  | 2014-2016 | 2850 | 0.80 |
| **≥5 vs. ≥10 µg/dL** | 2017-2018 | 2823 | 0.84 |
|  | 2014-2016 | 2850 | 0.85 |

^a^N=total number of census tracts included in the analysis

^b^ 0.41-0.6 indicates moderate agreement; 0.61-0.8 indicates substantial agreement; and 0.81-0.99 indicates near perfect agreement

**Table S-2. Agreement between different blood Pb reference values for two time periods using the top 20^th^ percentile method (children 0 to <6 years old)**

| **Blood Pb Reference Values** | **Period** | **N^a^** | **Kappa^b^** |
| --- | --- | --- | --- |
| **≥3.5 vs. ≥5 µg/dL** | 2017-2018 | 2823 | 0.81 |
|  | 2014-2016 | 2850 | 0.84 |
| **≥3.5 vs. ≥10 µg/dL** | 2017-2018 | 2823 | 0.60 |
|  | 2014-2016 | 2850 | 0.62 |
| **≥5 vs. ≥10 µg/dL** | 2017-2018 | 2823 | 0.63 |
|  | 2014-2016 | 2850 | 0.66 |

^a^N=total number of census tracts included in the analysis

^b^ 0.41-0.6 indicates moderate agreement; 0.61-0.8 indicates substantial agreement; and 0.81-0.99 indicates near perfect agreement

Table S-3. Statistical results of Getis-Ord Gi* geospatial analysis for different periods using 3.5 µg/dL as blood Pb reference value for EBLL (children aged 0 to <6 years old)

|  | Total # Census Tracts | Kappa^a^ |
| --- | --- | --- |
| Exceedance Rate of EBLLs ≥3.5 µg/dL: 2014-2016 vs. 2005-2007 | 2656 | 0.70 |
| Exceedance Rate of EBLLs ≥3.5 µg/dL: 2014-2016 vs. 2008-2010 | 2702 | 0.77 |
| Exceedance Rate of EBLLs ≥3.5 µg/dL: 2014-2016 vs. 2011-2013 | 2733 | 0.85 |
| Exceedance Rate of EBLLs ≥3.5 µg/dL: 2014-2016 vs. 2017-2018 | 2809 | 0.84 |

^a^ 0.41-0.6 indicates moderate agreement; 0.61-0.8 indicates substantial agreement; and 0.81-0.99 indicates near perfect agreement

Table S-4. Statistical results of top 20^th^ percentile census tracts for different periods using 3.5 µg/dL as blood Pb reference value for EBLL (children aged 0 to <6 years old)

|  | Total # Census Tracts | Kappa^a^ |
| --- | --- | --- |
| Exceedance Rate of EBLLs ≥3.5 µg/dL: 2014-2016 vs. 2005-2007 | 2656 | 0.63 |
| Exceedance Rate of EBLLs ≥3.5 µg/dL: 2014-2016 vs. 2008-2010 | 2702 | 0.69 |
| Exceedance Rate of EBLLs ≥3.5 µg/dL: 2014-2016 vs. 2011-2013 | 2733 | 0.74 |
| Exceedance Rate of EBLLs ≥3.5 µg/dL: 2014-2016 vs. 2017-2018 | 2809 | 0.69 |

^a^ 0.41-0.6 indicates moderate agreement; 0.61-0.8 indicates substantial agreement; and 0.81-0.99 indicates near perfect agreement

Table S-5. Urban and rural census tracts and population by census tract hotspot method using 3.5 µg/dL as blood Pb reference value (children aged 0 to <6 years old)

| Identification Method | | Number of Census Tracts | | | Total Population 0 to <6 Years Old | | |
| --- | --- | --- | --- | --- | --- | --- | --- |
|  |  | Urban | Rural | Ratio | Urban | Rural | Ratio |
| Getis-Ord Gi* | Non-hotspot | 1971 | 524 | 3.8 | 621,008 | 160,585 | 3.9 |
| *Getis-Ord Gi** | *Hotspot* | *353* | *2* | *177* | *77,832* | *541* | *144* |
| Top 20^th^ Percentile | Non-hotspot | 1788 | 492 | 3.6 | 574,616 | 152,932 | 3.8 |
| *Top 20^th^ Percentile* | *Hotspot* | *536* | *34* | *16* | *124,224* | *8,194* | *15* |

Table S-6. Statistical Agreement for the Getis-Ord Gi* method (EBLL using 3.5 µg/dL) with EJSCREEN 2017 Pb Paint EJ Index, Schultz et al., 2017 Regression Model Approach BLL Data, HUD Deteriorated Paint Index (children aged 0 to <6 years old)

|  | **Total # Census Tracts** | **Sensitivity** | **Specificity** | **Kappa^a^** |
| --- | --- | --- | --- | --- |
| EBLL 2014-2016 vs. EJSCREEN | 2850 | 77 | 91 | 0.58 |
| EBLL 2014-2016 vs. Schultz | 2850 | 87 | 91 | 0.64 |
| EBLL 2014-2016 vs. HUD | 2299 | 72 | 90 | 0.54 |
|  |  |  |  |  |
| EJSCREEN vs. Schultz | 2952 | N/A | N/A | 0.82 |
| EJSCREEN vs. HUD | 2372 | N/A | N/A | 0.79 |
| Schultz vs. HUD | 2372 | N/A | N/A | 0.75 |

^a^ 0.41-0.6 indicates moderate agreement; 0.61-0.8 indicates substantial agreement; and 0.81-0.99 indicates near perfect agreement
